# Accurate prediction of huanglongbing occurrence in citrus plants by machine learning-based analysis of symbiotic bacteria

**DOI:** 10.3389/fpls.2023.1129508

**Published:** 2023-05-29

**Authors:** Hao-Qiang Liu, Ze-long Zhao, Hong-Jun Li, Shi-Jiang Yu, Lin Cong, Li-Li Ding, Chun Ran, Xue-Feng Wang

**Affiliations:** ^1^ Citrus Research Institute, Southwest University/Chinese Academy of Agricultural Sciences, National Engineering Research Center for Citrus, Chongqing, China; ^2^ Shanghai BIOZERON Biotechnology Co., Ltd., Shanghai, China

**Keywords:** citrus microbiome, Huanglongbing, machine learning, meta-analysis, community assembly

## Abstract

Huanglongbing (HLB), the most prevalent citrus disease worldwide, is responsible for substantial yield and economic losses. Phytobiomes, which have critical effects on plant health, are associated with HLB outcomes. The development of a refined model for predicting HLB outbreaks based on phytobiome markers may facilitate early disease detection, thus enabling growers to minimize damages. Although some investigations have focused on differences in the phytobiomes of HLB-infected citrus plants and healthy ones, individual studies are inappropriate for generating common biomarkers useful for detecting HLB on a global scale. In this study, we therefore obtained bacterial information from several independent datasets representing hundreds of citrus samples from six continents and used these data to construct HLB prediction models based on 10 machine learning algorithms. We detected clear differences in the phyllosphere and rhizosphere microbiomes of HLB-infected and healthy citrus samples. Moreover, phytobiome alpha diversity indices were consistently higher for healthy samples. Furthermore, the contribution of stochastic processes to citrus rhizosphere and phyllosphere microbiome assemblies decreased in response to HLB. Comparison of all constructed models indicated that a random forest model based on 28 bacterial genera in the rhizosphere and a bagging model based on 17 bacterial species in the phyllosphere predicted the health status of citrus plants with almost 100% accuracy. Our results thus demonstrate that machine learning models and phytobiome biomarkers may be applied to evaluate the health status of citrus plants.

## Introduction

The plant phytobiome, consisting of the rhizosphere, phyllosphere, and endosphere, harbors diverse microbes that affect plant growth and health (Berendsen et al., 2012; Liu et al., 2019; Liu et al., 2020). Considerable research has been carried out on the diversity, composition, and function of the phytobiome of various plant species, including rice (Edwards et al., 2015), maize (Peiffer et al., 2013), corn (Jat et al., 2021), soybean (Zhang et al., 2018), cucumber (Zhou et al., 2022a), citrus (Xu et al., 2018), and tomato (Oyserman et al., 2022). Microbes inhabiting plant surfaces or internal tissues produce metabolites that support plant growth by regulating physiological processes (e.g., nutrient absorption and pathogen suppression) (Trivedi et al., 2020). The identification of microbes associated with specific phenotypes is currently a fundamental objective of phytobiome researchers. Moreover, changes to the plant microbiome are influenced by diverse biotic and abiotic factors of the host and surrounding environment (Dastogeer et al., 2020). Niche and neutral theory-based approaches have been used to explore the mechanisms modulating the microbiome assembly, with all factors classified into deterministic or stochastic processes (Chen et al., 2019). These studies have expanded our understanding of phytobiomes, with implications for managing plant-associated microbiomes to enhance crop production (French et al., 2021).

Citrus is an economically important fruit crop comprising several widely cultivated species initially domesticated more than 1,000 years ago (Gmitter and Hu, 1990). Because their fruits contain an abundance of diverse nutrients, vitamins, and dietary fiber, citrus species are extensively cultivated worldwide, with annual yields exceeding 120 million tons (Mahato et al., 2021). Nevertheless, citrus production has been restricted by global climate change and the prevalence of diseases (Wang, 2019; Wang, 2020). Huanglongbing (HLB), a fatal disease, is the biggest threat to citrus production. The high prevalence of HLB and associated, considerable yield losses have recently renewed interest in this disease (Das et al., 2019). HLB leads to a loss of citrus root carbonaceous compounds, malfunctioning phloem tissues, and decreased release of photosynthates, all of which impair the transport of photoassimilates (Trivedi et al., 2020). In addition, HLB upsets the nutrient balance by altering the ability of roots to absorb and transport nutrients and water (Wang, 2020). HLB is mainly caused by the bacterium *Candidatus Liberibacter asiaticus* (CLas) (Li et al., 2017). Because CLas cannot be monocultured (Killiny-Mansour, 2019), clarifying citrus-associated microbiome dynamics due to HLB pressure is of serious interest.

A comprehensive understanding of citrus-associated microbiomes may lead to the development of sustainable, environmentally friendly methods for increasing citrus plant health and productivity. Global patterns in citrus phytobiomes are already being revealed (Xu et al., 2018; Zhang et al., 2021; Penyalver et al., 2022). For instance, the International Citrus Microbiome Consortium, established in 2015, has performed sequencing analyses of citrus rhizosphere and soil microbiome samples from major citrus-producing regions on six continents (Wang et al., 2015). In addition, recent Illumina-based sequencing of 16S rRNA genes has revealed that HLB alters the microbiome of citrus rhizospheres and phyllospheres (Srivastava et al., 2022). Most related research has only focused on healthy citrus microbiomes or HLB-infected ones, however, with relatively few comparative studies performed on both types of microbiomes (Li et al., 2017; Ginnan et al., 2018; Blacutt et al., 2020). Whether some citrus taxa in worldwide cultivation are robust and universally responsive to HLB remains unclear.

In this study, we systematically reviewed available data on the citrus phytobiome and compared the bacterial communities of healthy and HLB-infected citrus plants. In addition, machine learning approaches were applied to identify potential biomarkers for HLB occurrence after technical biases, geographic distribution, and tissue specificity were taken into account. These analyses allowed us to reveal the diversity, composition, and mechanisms underlying the bacterial community assembly of HLB-infected citrus plants. Finally, we developed a phytobiome-based model to predict HLB outbreaks under field conditions.

## Materials and methods

### Data collection and description

**Figure 1:**
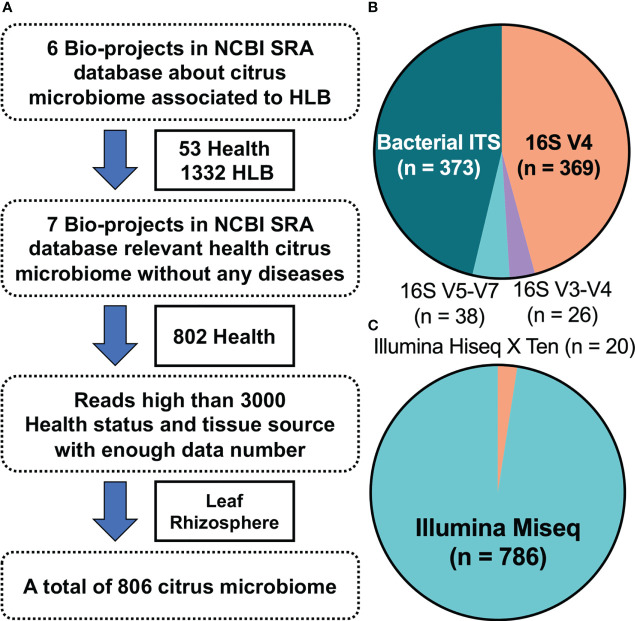
Study characteristics. **(A)** Thirteen bio-projects were considered, from which 806 bacterial samples were ultimately selected. **(B)** Details regarding the amplified regions for all selected samples. **(C)** Ratio of the sequencing platforms for all selected samples.

Screening of the National Center for Biotechnology Information Sequence Read Archive database using “citrus” and “huanglongbing” as keywords yielded six HLB-related citrus microbiome bio-projects that included 1,385 bacterial samples (53 healthy citrus samples and 1,332 HLB-infected ones). Another seven bio-projects with 802 bacterial samples from healthy citrus plants were also considered. The metadata for these bio-projects (**Table S1**) were classified according to source tissue/material into five groups (**Table S2**). Because samples from budwood, bulk soil, and attached insects were limited, only leaf and rhizosphere datasets were analyzed further (**Table S2**). Moreover, samples with fewer than 3,000 reads were removed to eliminate abnormal sequencing results. Finally, 806 citrus microbiome samples were retained (**Figure 1A**): 29 and 207 from healthy citrus leaves and rhizospheres, respectively, and 267 and 303 from HLB-infected citrus leaves and rhizospheres, respectively (**Table S3**). Four amplified regions, mainly bacterial ITS (46.28%) and 16S V4 (45.78%), were identified in the metadata (**Figure 1B**). The sequencing data were mostly produced on the Illumina MiSeq platform, with only 20 samples sequenced using the Illumina HiSeq X Ten system (**Figure 1C**).

### Data processing

The collected datasets, in FASTQ format, were analyzed using a standard Quantitative Insights Into Microbial Ecology 2 (QIIME2) pipeline (Bolyen et al., 2019). After removal of primer sequences and quality control, the resulting clean reads were clustered to obtain amplicon sequence variants (ASVs) using the DADA2 plug-in unit (Bokulich et al., 2018). Each ASV was assigned to a taxon according to a closed-reference strategy using the SILVA database (release 138) (Yilmaz et al., 2014). In this approach, a reference database comprising the full-length sequences of amplified targets was predefined and used to generate representative sequences and taxonomically classify sequences produced by different primers (Yu et al., 2018). Non-bacterial ASVs (i.e., chloroplasts and archaea) and singletons (ASVs with only one read) were discarded. Finally, the ASV abundance tables were rarefied to 3,000 reads per sample because of the unequal sequencing depth.

### Statistical analyses

All statistical analyses were conducted in R (v4.0.2), and the results were visualized using the R “ggplot2” package (R Core Team, 2020). The following three alpha diversity indices for bacterial communities were calculated using the “vegan” package (Oksanen et al., 2020): Chao1 (richness), Shannon’s (diversity), and Pielou’s J (evenness). Differences in the alpha diversity and relative abundance of bacterial phyla and genera between healthy and HLB-infected citrus leaf and rhizosphere samples were analyzed using the *t*-test function. Bray–Curtis distances between bacterial communities were calculated using the vegdist function in the “vegan” package. These distances were then used in a principal coordinate analysis (PCoA) with the pcoa function in the “ape” package and a permutational multivariate analysis of variance (PERMANOVA) with the adonis function in the “vegan” package to evaluate differences in the bacterial communities of healthy and HLB-infected citrus leaf and rhizosphere samples. A Venn diagram-based analysis was performed using the “VennDiagram” package (Chen, 2022) to identify shared bacterial ASVs among samples. Differences in the total relative abundance of shared ASVs were assessed by ANOVA followed by Tukey’s HSD test (“multcomp” package). Finally, a neutral community model was used to determine the potential importance of deterministic and stochastic processes on the assembly of bacterial communities. In this model, two variables were defined: *m*, an estimate of the dispersal between communities, and *R^2^*, which represented the ratio of the contributions of stochastic processes (Sloan et al., 2006).

### Machine-learning modeling

To more precisely distinguish between bacterial communities of HLB-infected and healthy citrus plants, we applied the following 10 established machine learning algorithms (Gupta et al., 2021) to construct models according to relative abundances of bacteria (phylum to species levels): logistic regression, decision tree, *k*-nearest neighbor, bagging, gradient boosting, Bayes classification, artificial neural network, conditional inference tree, random forests, and support vector machines. Models were constructed for leaf and rhizosphere microbiomes separately. More specifically, 70% of healthy and HLB-infected citrus samples were randomly selected as training data to construct models, and the remaining 30% were used as testing data for model validation. The predicted results were compared with actual health status using two metrics: receiver operating characteristic curve (ROC) and area under the curve (AUC) scores (Sing et al., 2005). Finally, the best-performing models (i.e., those with the highest AUC scores and accuracies) were identified, and the importance of various features in the classification was determined.

## Results

### Differences in the microbiome diversity of HLB-infected and healthy citrus samples

**Figure 2:**
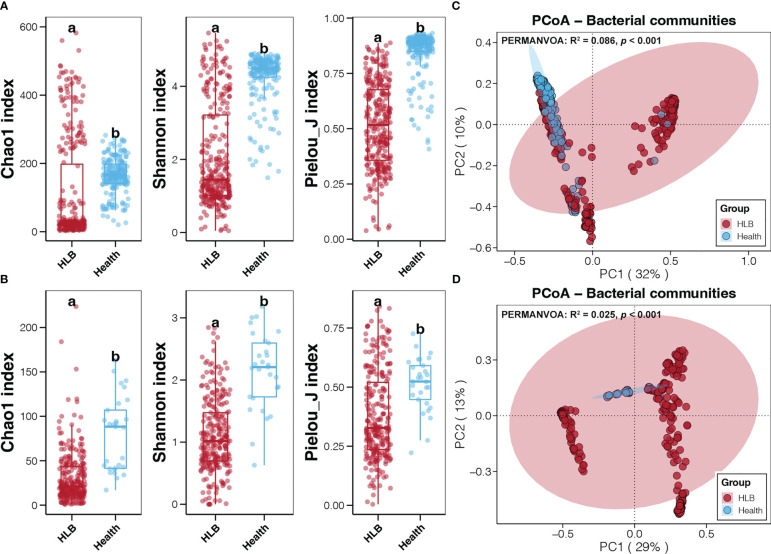
Differences in the alpha diversity indices for the bacterial communities of the HLB-infected and healthy citrus leaf **(A)** and rhizosphere **(B)** samples. Different lowercase letters above each box in the same subfigure represent significant differences between groups (Student’s *t*-test, *p* < 0.05). Results of the PCoA and PERMANOVA conducted on the basis of the Bray-Curtis distance for the bacterial communities of the HLB–infected and healthy citrus leaves **(C)** and rhizospheres **(D)**.

Meta-analysis of sequencing data for 806 bacterial samples from six continents generated a merged bacterial ASV table comprising more than 3,700 taxa. All of these bacterial ASVs were annotated at the phylum level, but only 67.45% and 25.5% were annotated at genus and species levels, respectively (**Figure S1**). We rarefied the sequencing data to 3,000 reads per sample before calculating alpha diversity indices. which were lowest and highest for HLB-infected citrus leaf and healthy citrus rhizosphere microbiomes, respectively (**Figure S2**). More importantly, Chao1, Shannon’s, and Pielou’s J indices of leaf and rhizosphere bacterial communities were significantly lower for HLB-infected citrus samples than healthy ones (Student’s *t*-test, *p* < 0.05; **Figures 2A, B**). In addition, inter-individual differences in the alpha diversity indices of leaf and rhizosphere bacterial communities were more obvious for HLB-infected samples than for healthy samples (**Figures 2A, B**). According to PCoA and PERMANOVA, the bacterial community structures of HLB-infected and healthy citrus leaf and rhizosphere samples were significantly different (*p* < 0.05; **Figures 2C, D**). Moreover, inter-individual differences in leaf and rhizosphere bacterial communities were greater for HLB-infected samples than for healthy ones (**Figures 2C, D**), consistent with the differences observed in alpha diversity indices.

### Taxonomic classification of bacteria in citrus leaf and rhizosphere microbiomes

**Figure 3:**
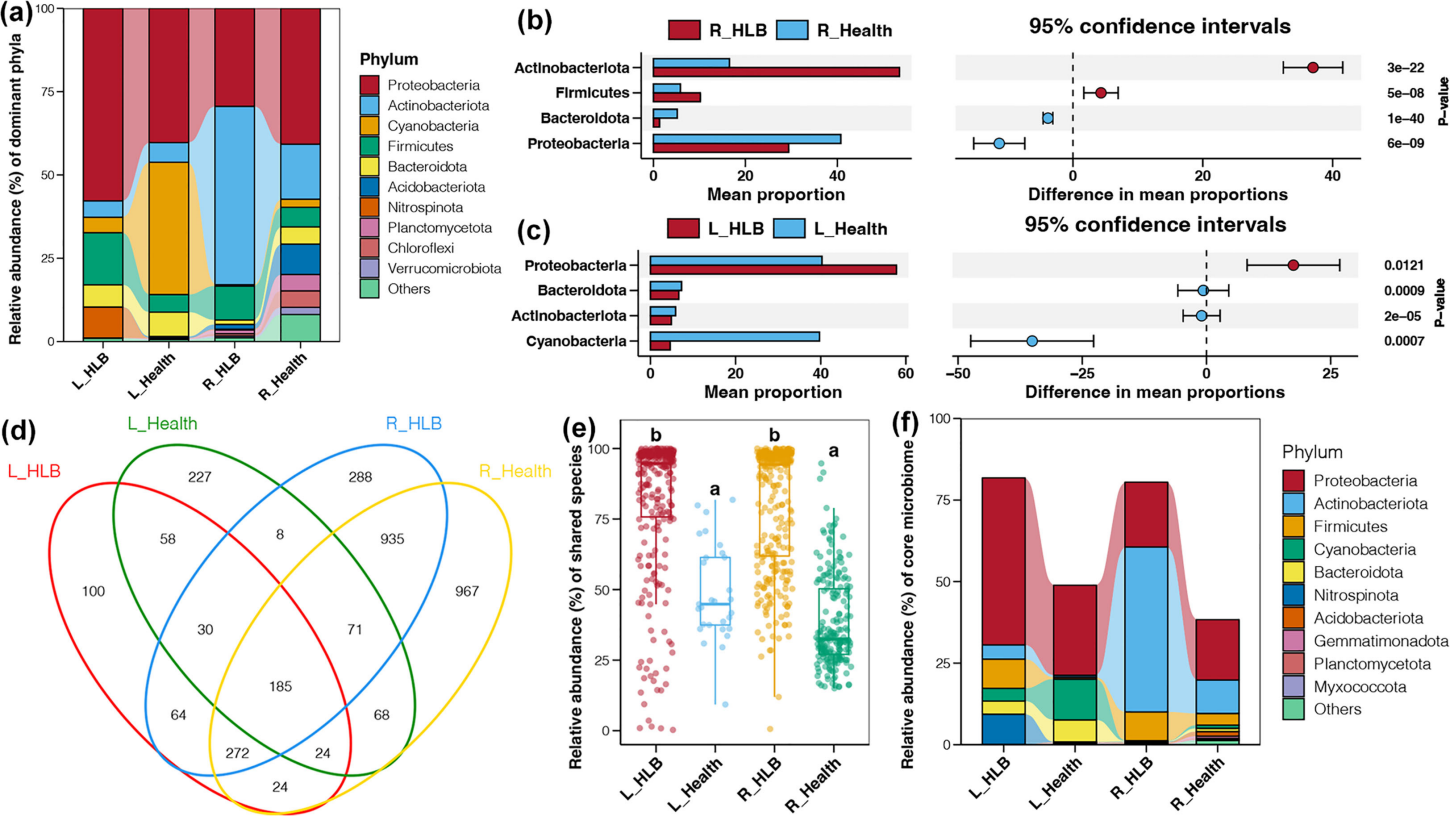
**(A)** Relative abundance (%) of the major phyla present in the bacterial communities in the HLB-infected and healthy citrus leaves and rhizospheres. Bacterial phyla with significantly different relative abundances in the HLB-infected and healthy citrus rhizosphere **(B)** and leaf **(C)** samples. **(D)** Venn diagram of the number of shared ASVs in the HLB-infected and healthy citrus leaves and rhizospheres. **(E)** Differences in the total relative abundances of shared bacterial taxa in the HLB-infected and healthy citrus leaves and rhizospheres. Different lowercase letters above each box in the same subfigure represent significant differences between groups (Tukey’s HSD test, *p* < 0.05). **(F)** Relative abundance (%) of the shared bacterial phyla in the HLB–infected and healthy citrus leaves and rhizospheres.

The most dominant bacterial phylum in citrus leaf and rhizosphere samples was Proteobacteria, which was followed by Cyanobacteria and Actinobacteriota in leaf and rhizosphere microbiomes, respectively (**Figure 3A**). Actinobacteriota and Firmicutes were more abundant in HLB-infected citrus rhizospheres than in healthy citrus rhizospheres, whereas the opposite pattern was observed for Proteobacteria and Bacteroidota (**Figure 3B**). HLB-infected leaves had a higher relative abundance of Proteobacteria, whereas healthy leaves had higher relative abundances of Cyanobacteria, Actinobacteriota, and Bacteroidota (**Figure 3C**). We also detected 186 shared taxa among bacterial communities (**Figure 3D**). The total relative abundances of shared bacteria in citrus leaf and rhizosphere microbiomes were significantly higher for HLB-infected samples than for healthy ones (Tukey’s HSD test, *p* < 0.05; **Figure 3E**). Most shared bacteria with increased abundances in HLB-infected samples belonged to Proteobacteria, Firmicutes, and Nitrospinota (leaf microbiome) or Actinobacteriota (rhizosphere microbiome) (**Figure 3F**).

### Analysis of microbiome assembly mechanisms of HLB-infected and healthy citrus samples

**Figure 4:**
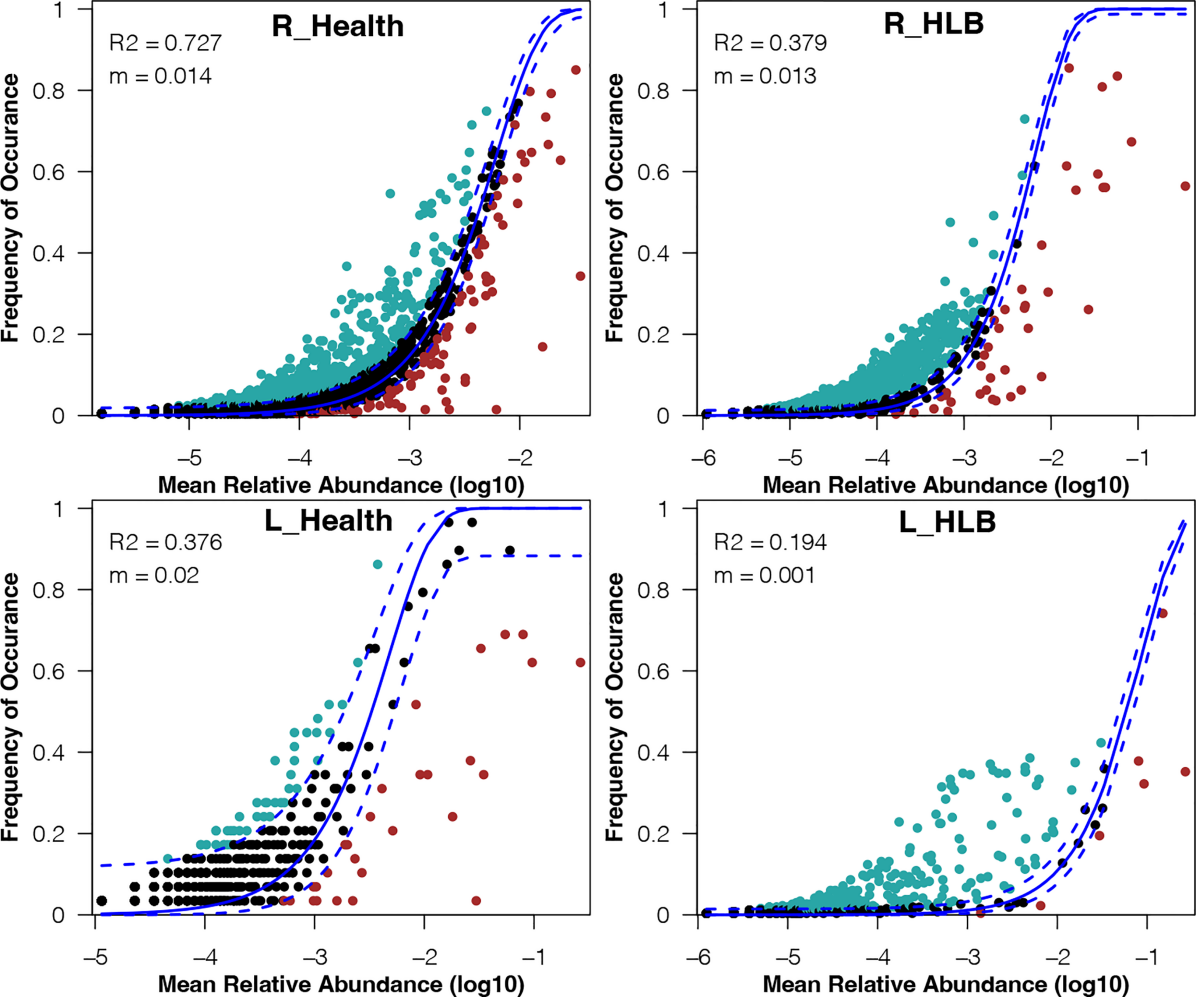
Fit of the neutral community model for the bacterial communities in the HLB-infected and healthy citrus leaves and rhizospheres. The solid and dashed lines indicate the best fit to the neutral community model and the 95% confidence intervals for the model predictions, respectively. m, meta–community size times immigration; R^2^, how well the data fit the model.

To explore the mechanisms underlying the differences in leaf and rhizosphere microbiome assemblies between HLB-infected and healthy citrus samples, we examined the relative effects of niche and neutral processes on the assembly of bacterial communities. The neutral community model explained a large proportion of the variance in the bacterial community of healthy citrus rhizospheres (*R* ^2 =^ 0.727), whereas only 37.9% of the corresponding variance in HLB-infected citrus rhizospheres was explained by this model (**Figure 4**). In contrast, the neutral community model explained only 19.4% and 37.6% of the variance in bacterial communities of HLB-infected and healthy citrus leaves, respectively (**Figure 4**). These results indicate that the bacterial community assembly in healthy citrus rhizospheres and leaves was respectively governed by stochastic and deterministic processes. More importantly, HLB obviously decreased the contribution of stochastic processes to the assembly of bacterial communities in citrus leaves and rhizospheres.

### Bacterial communities useful for distinguishing between HLB-infected and healthy citrus samples

**Figure 5:**
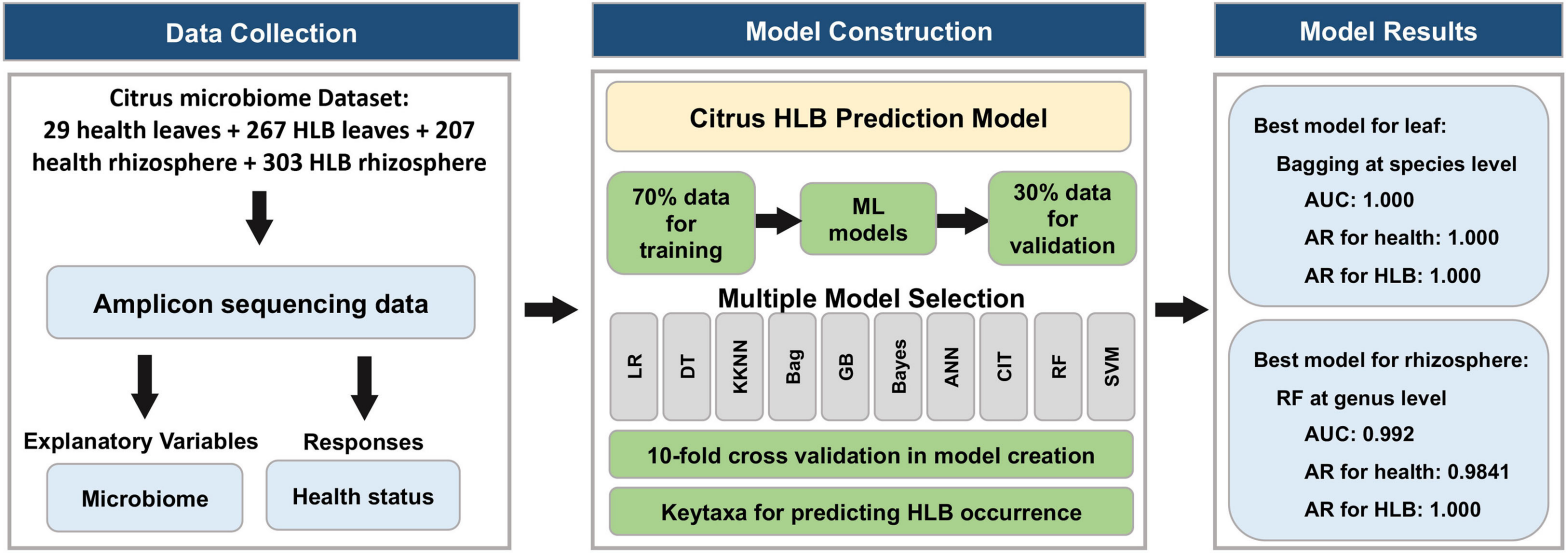
Steps involved in generating and validating the health status prediction models. Ten different classification algorithms were used for predicting HLB infections. Model prediction performances were evaluated on the basis of bacterial abundances from the phylum to species levels.

To determine whether the properties of leaf and rhizosphere bacterial communities may be useful biomarkers for distinguishing between HLB-infected and healthy citrus plants, we constructed 10 machine learning models. The accuracy of model predictions based on the testing data as well as AUC and ROC data derived from the models were used to evaluate model performance (**Figures S3**, **S4**). We also used the accuracy of predictions for healthy and HLB-infected samples to select the best models (**Figure S5**). The bagging model trained at the species level and the random forest model trained at the genus level were found to be the best models for classifying leaf and rhizosphere samples, respectively (**Figure 5**).

**Figure 6:**
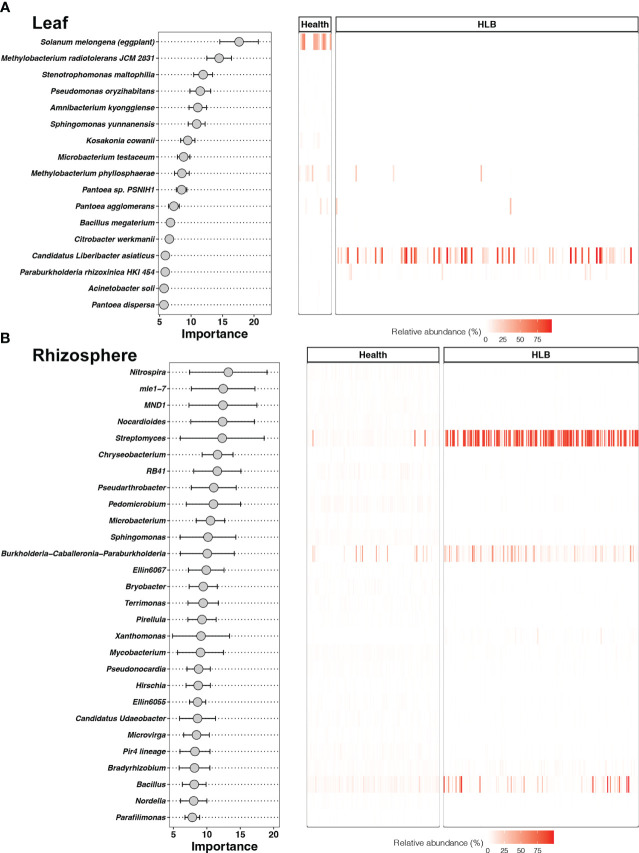
Biomarker taxa ranked in descending order of importance for the accuracy of predictions and their relative abundances in the leaf **(A)** and rhizosphere **(B)** models.

The bagging model for detecting HLB based on bacterial species in citrus leaves was constructed using 17 species—the most important of which was *Solanum melongena* (eggplant) (**Figure 6A**). Two of these species, (CLas and *Paraburkholderia rhizoxinica* HKI 454), had higher relative abundances in HLB-infected leaves, whereas 15 species had higher relative abundances in healthy leaves (**Table S4**). In terms of the citrus rhizosphere, 28 bacterial genera were defined as biomarker taxa for HLB, of which *Nitrospira* was the most important (**Figure 6B**). Four of these genera had higher relative abundances in HLB-infected citrus rhizospheres, and the other 24 genera had higher relative abundances in healthy citrus rhizospheres (**Table S5**). *Streptomyces*, *Burkholderia-Caballeronia-Paraburkholderia*, and *Bacillus* were all enriched in HLB-infected citrus rhizospheres to the same degree relative to healthy rhizospheres.

## Discussion

The exploration of biomarkers common to the phytobiome of citrus plants infected with HLB is critical for developing improved methods for diagnosing plant diseases caused by bacteria and for determining optimal treatments. Nevertheless, knowledge of whether certain microbial lineages consistently respond to HLB across global biogeographic regions is unclear. In this study, we performed a meta-analysis of HLB-infected and healthy citrus rhizosphere and phyllosphere microbiomes on a global scale to screen for biomarkers useful for detecting HLB. By combining global datasets, we determined that Proteobacteria, Actinobacteria, Acidobacteria, and Bacteroidetes were the predominant bacterial phyla in healthy citrus rhizospheres (**Figure 3A**). This result is consistent with the findings of an earlier study on citrus rhizosphere samples from six continents (Xu et al., 2018). Furthermore, the dominant bacterial phyla detected in healthy citrus phyllospheres as well as the distribution of their relative abundances in this study are in accordance with published results (Blaustein et al., 2017; Bai et al., 2019; Wu et al., 2020). These observations revealing similarities in citrus phytobiomes from various geographical regions imply that host phylogeny may influence phytobiome assembly more than geographical factors. Recent reports have described adaptive matching between rhizosphere and phyllosphere microbiomes and plant hosts (Lajoie et al., 2020; Escudero-Martinez et al., 2022).

Symbiotic microbiome homeostasis is closely associated with host physiological features and health (Paasch and He, 2021). As a fundamental indicator of the stability and performance of microbial communities, diversity is a crucial index for phytobiomes (Sare et al., 2020). A decrease in phytobiome richness and diversity is often responsible for the increased susceptibility of plant hosts to potentially harmful factors (Agler et al., 2016). In addition, decreased phytobiome diversity may be due to insufficient competition between resident commensals and invading pathogens (Thoms et al., 2021). In the current study, alpha diversity decreased in the rhizosphere and phyllosphere microbiomes of citrus plants infected with HLB (**Figures 2A, B**). Alpha diversity indices varied considerably among HLB-infected samples, however, which may be related to the features of the sequenced regions and the methods used. Moreover, the PERMANOVA results revealed that data source and sequenced target region had larger effects on citrus phytobiome composition than did citrus health status, geographic location, and tissue source (**Table S6**). Alpha diversity and citrus phytobiome composition were thus strongly affected by the methods used in different bio-projects and may therefore not be robust indicators of citrus health status.

Most research conducted on a global scale has suggested that relatively few bacterial taxa represent a large proportion of highly diverse bacterial communities. For example, a study examining global soil samples found that 2% of bacterial taxa accounted for nearly half of bacterial communities at various sites (Delgado-Baquerizo et al., 2018). Notably, Xu et al. (2018) reported that a small number of bacterial taxa (< 10%) were the core taxa in rhizospheres of citrus samples collected on various continents. In the present study, we detected 138 bacterial taxa common to all samples; these taxa represented approximately half of the bacterial communities in healthy citrus samples (**Figure 3E**). In contrast, the median total abundances of shared bacterial taxa in HLB-infected citrus leaves and rhizospheres corresponded to approximately 95% of the entire communities (**Figure 3F**). Hence, a limited number of core taxa were associated with the occurrence of HLB in citrus plants. This result may help to explain the decrease in alpha diversity index values among HLB samples.

The mechanisms mediating the assembly of phytobiome communities must be considered when designing plant microbiome management strategies (Trivedi et al., 2020). From a meta-community perspective, bacterial community assembly is governed by both deterministic and stochastic processes (Tian et al., 2022). If communities are controlled by deterministic processes, species will occupy specific ecological niches in a predictable fashion (Vanwonterghem et al., 2014). In contrast, multiple species can exist in similar or overlapping habitats in communities affected by stochastic fluctuations (Sloan et al., 2006). Our findings suggest that stochastic and deterministic processes are critical for shaping healthy citrus rhizosphere and phyllosphere microbiomes (**Figure 4**). Inconsistencies between citrus rhizosphere and phyllosphere community assemblies may be attributed to their different lifestyles and functions. Plant leaves and roots are located above- and belowground, respectively, which allows phyllosphere and rhizosphere microbiomes to perform different functions (Trivedi et al., 2012). In addition, low nutrient levels and long-term illumination may lead to changes in the abundances of specific bacteria in the phyllosphere microbiome (Carvalho and Castillo, 2018), thereby increasing the importance of deterministic processes. Moreover, we observed that deterministic processes mediated phytobiome assembly more substantially in HLB-infected citrus samples than in healthy ones (**Figure 4**). Similar observations have been reported for phytobiomes of other diseased plants and the gut microbiota of diseased animals (Yao et al., 2018; Liu et al., 2022; Zhang et al., 2022). HLB is a disease caused by pathogenic bacteria, and the enriched pathogens will decrease the abundance of species with an overlapping niche and select for species without niche conflicts through competition (Chase, 2011). This phenomenon may explain why deterministic processes shaped the HLB-infected citrus microbiome.

Among the many statistical methods for elucidating the complex relationships between microbial communities and specific phenotypes, machine learning-based methods are considered the most promising (Torija and Ruiz, 2015). Machine learning approaches take various forms according to their algorithms (e.g., unsupervised, semi-supervised, or supervised learning) (Ghannam and Techtmann, 2021). In the present study, we assessed the relationship between the health status of citrus samples collected worldwide and the relative abundance of bacteria at different taxonomic levels in the rhizosphere and phyllosphere. We used 10 machine learning approaches and found that the most appropriate models for predicting HLB infections were supervised learning methods (random forest and bagging) that were based on rhizosphere and phyllosphere bacterial genera and species (**Figure 5**). A previous meta-analysis demonstrated that supervised learning models for soil microbiomes may be used to predict the potential occurrence of *Fusarium* wilt disease in plants (Yuan et al., 2020). In addition, supervised learning methods have identified robust and reproducible features relevant for diagnosing shrimp diseases according to meta-analyses of gut microbiota (Sha et al., 2022). The origin and quality of sea cucumber cultured in diverse geographic regions has also been accurately predicted using random forest models for gut microbiota (Zhao et al., 2022). Furthermore, supervised machine learning approaches have accurately predicted environmental health variables following analyses of microbiome data (Zhou et al., 2022b). Random forest models for microbial communities have predicted the soil health parameters of agroecosystems, with accuracies exceeding 80% (Wilhelm et al., 2022). These results provide convincing evidence of the utility of supervised learning methods for establishing models that accurately predict the health status of plants.

The vector of HLB, a bacterial infection of citrus trees, is believed to be the Asian citrus psyllid *Diaphorina citri* (Galdeano et al., 2020). At present, the dominant control strategies for HLB are removal of HLB-symptomatic citrus trees and the spraying of insecticides to restrict the psyllid (Coletta-Filho et al., 2014). Because HLB-infected trees may remain asymptomatic for several months, however, the efficacy of current disease control measures is limited (Lee et al., 2015). Using the bagging model, we identified crucial bacterial taxa related to citrus HLB disease incidence, including CLas and *Paraburkholderia rhizoxinica* in the phyllosphere and *Streptomyces*, *Burkholderia-Caballeronia-Paraburkholderia*, and *Bacillus* in the rhizosphere. A recent study indicated that CLas is the main pathogen responsible for HLB outbreaks (Ginnan et al., 2020). *Paraburkholderia rhizoxinica* is an endofungal bacterium that has a symbiotic relationship with phytopathogenic fungi (Braga et al., 2019). In contrast, bacteria enriched in HLB-infected citrus rhizospheres in our study were not directly related to the disease phenotype. These bacteria included antibiotic producers and species with detrimental effects on community stability (Nicholson, 2002; de Lima et al., 2012). Our findings imply that the risk of HLB can be assessed by screening for a few specific known pathogens in citrus leaves. Data for only 29 healthy citrus phyllospheres were included in our analyses, however, and a limited sample size and bias between two classifications can cause machine learning models to overestimate the risks of HLB outbreaks (Gupta et al., 2021). We thus recommend the use of a random forest model based on bacterial genera in the rhizosphere to predict the likelihood of HLB in citrus plants.

## Conclusions

In this study, we analyzed the utility of phytobiome examinations for detecting HLB-infected citrus plants on a global scale. Meta-analyses involving the phytobiome data of hundreds of citrus samples revealed significant decreases in rhizosphere and phyllosphere microbiome diversities of HLB-infected samples relative to healthy ones. Furthermore, the onset of HLB increased the contribution of deterministic processes to citrus rhizosphere and phyllosphere microbiome assemblies. We also identified 17 and 28 HLB-related taxa in the phyllosphere and rhizosphere, respectively. These taxa may be exploited to accurately predict citrus HLB outbreaks on the basis of selected machine learning models. The findings of this study are relevant for evaluating the risks of HLB in citrus plants according to phytobiome compositions derived from 16S rRNA gene sequencing data. Advances in high-throughput sequencing technology and decreases in associated costs should enable researchers to further improve models for predicting the health status of agriculturally important plant species.

## Data availability statement

The datasets presented in this study can be found in online repositories. The names of the repository/repositories and accession number(s) can be found in the article/**Supplementary Material**.

## Author contributions

CR and X-FW: funding and project administration. H-QL: methodology, ideas, data curation, and statistical analysis. Z-LZ, H-JL, S-JY, LC and L-LD participated in this work. All authors contributed to the article and approved the submitted version.
