# Supplementary material for: Accurate prediction of huanglongbing occurrence in citrus plants by machine learning-based analysis of symbiotic bacteria
Source: Front Plant Sci. 2023 May 29;14:1129508. doi: 10.3389/fpls.2023.1129508 (PMC10258322; doi:10.3389/fpls.2023.1129508)
Supplement: Supplementary file 1 [file DataSheet_1.docx]

**Supporting information for**

#### **Accurately predicting huanglongbing occurrence in citrus plants according to a machine learning-based analysis of symbiotic bacteria**

**Figure S1.** Annotation of bacterial ASVs.

**
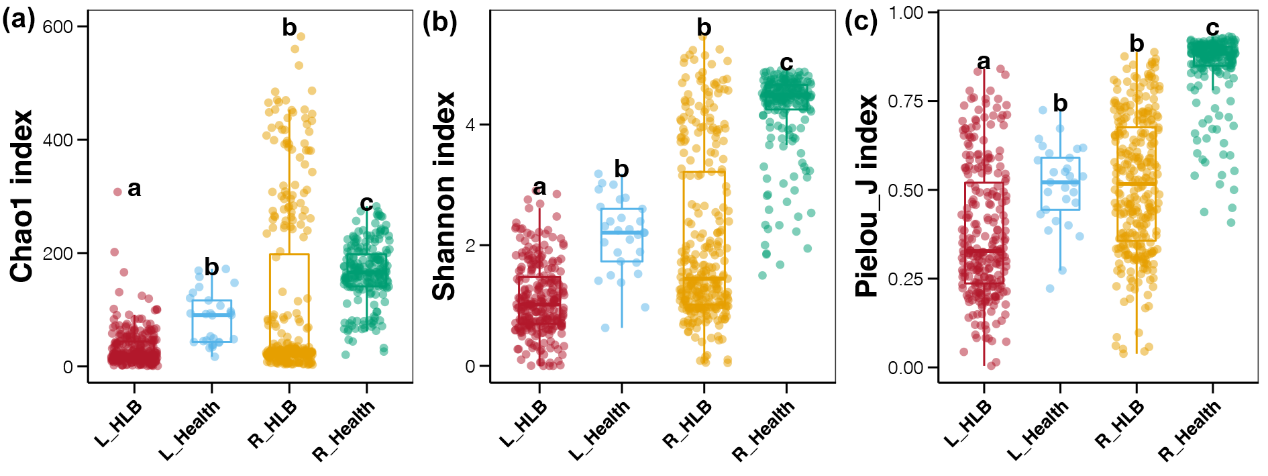
**

**Figure S2.** Differences in alpha diversity of bacterial communities among different groups. Different lowercase letters above each box in the same subﬁgure represent signiﬁcant differences between groups (Tukey’s HSD test, *p* < 0.05).


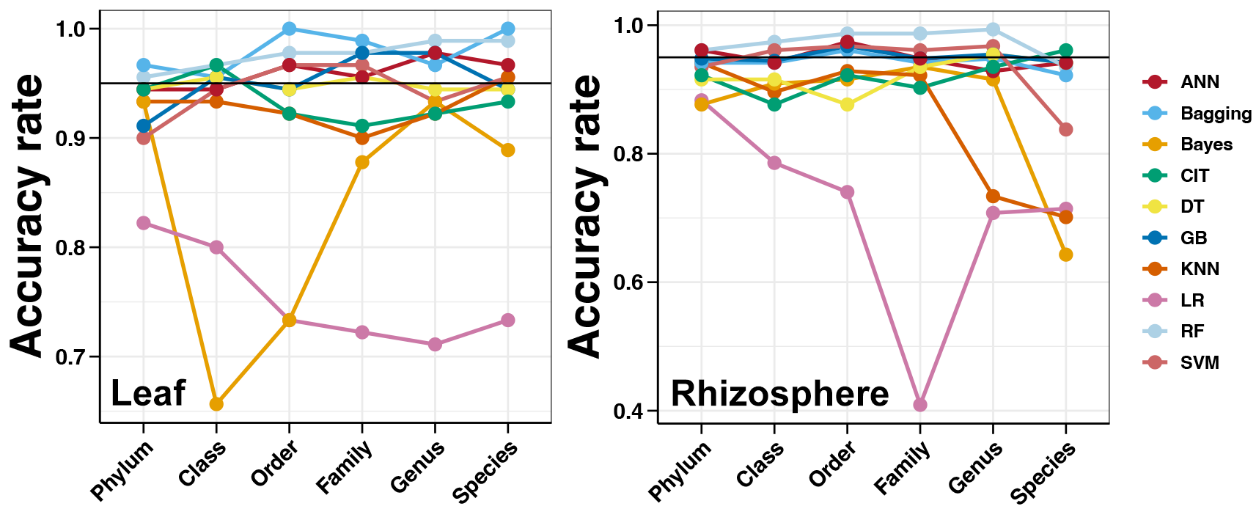


**Figure S3.** Accuracy rate of machine learning models.


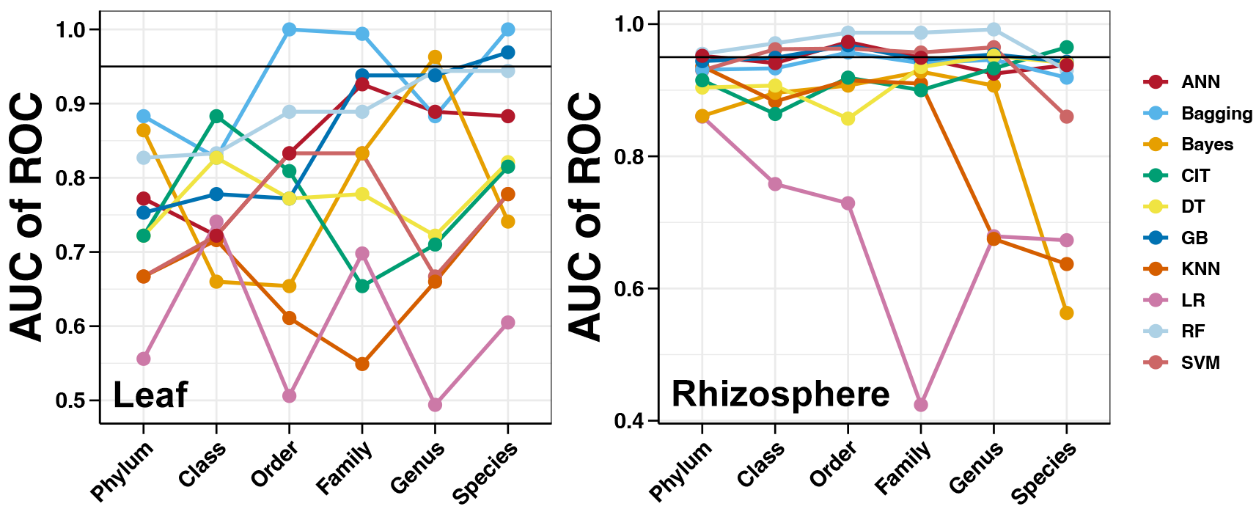


**Figure S4.** AUC value of machine learning models.


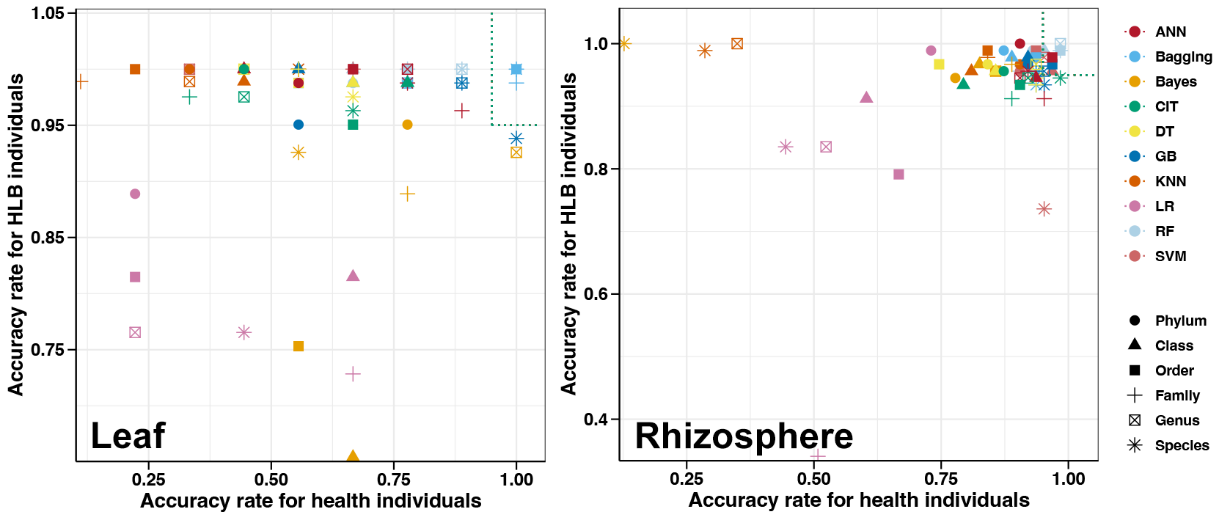


**Figure S5.** Accuracy rate for HLB and health samples of machine learning models.

**Table S1.** Sequence dataset of microbiome with citrus used in this meta-analysis.

| Study location | Host species | rRNA region | Sequencing platform | Accession number | References |
| --- | --- | --- | --- | --- | --- |
| Global | Not applicable | 16S V4 | Illumina MiSeq | PRJNA362455 | Xu et al., 2018 |
| Florida, USA | *Citrus sinensis L. Osbeck cv. Valencia* | 16S V4 | Illumina MiSeq | PRJNA362723 | Blaustein et al., 2017 |
| Hunan, China | Not applicable | 16S V4 | Illumina Hisq X Ten | PRJNA844183 | No publication information |
| USA | Lisbon lemon and Washington Navel orange | 16S V5-V7 | Illumina Miseq | PRJEB34710 | Padhi et al., 2019 |
| Florida, USA | Sour orange and Swingle citrumelo | ITS | Illumina MiSeq | PRJNA427826 | Ginnan et al., 2018 |
| Florida, USA | Not applicable | ITS | Illumina MiSeq | PRJNA546069 | Blacutt et al., 2020 |
| Florida, USA | Sour orange and Swingle citrumelo | ITS | Illumina MiSeq | PRJNA590541 | Ginnan et al., 2020 |
| Guangdong, China | Not applicable | 16S V3-V4 | Illumina MiSeq | PRJNA817184 | No publication information |
| Jiangxi, China | Not applicable | 16S V3-V4 | Illumina MiSeq | PRJNA669555 | Zhou et al., 2021 |
| Spain | *Citrus sinensis x Citrus trifoliata* | 16S V3-V4 | Illumina MiSeq | PRJEB15329 | Penyalver et al., 2022 |
| Iran | *Citrus sinensis* | 16S V1-V2 | Illumina MiSeq | PRJNA578610 | No publication information |
| Florida, USA | Not applicable | 16S V4 | Illumina MiSeq | PRJNA636132 | No publication information |
| Florida, USA | Not applicable | 16S V4 | Illumina MiSeq | PRJNA636781 | Castellano-Hinojosa et al., 2021 |

Xu, J., Zhang, Y., Zhang, P., Trivedi, P., Riera, N., Wang, Y., ... & Wang, N. (2018). The structure and function of the global citrus rhizosphere microbiome. Nature communications, 9(1), 1-10.

Blaustein, R. A., Lorca, G. L., Meyer, J. L., Gonzalez, C. F., & Teplitski, M. (2017). Defining the core citrus leaf-and root-associated microbiota: Factors associated with community structure and implications for managing huanglongbing (citrus greening) disease. Applied and environmental microbiology, 83(11), e00210-17.

Padhi, E. M., Maharaj, N., Lin, S. Y., Mishchuk, D. O., Chin, E., Godfrey, K., ... & Slupsky, C. M. (2019). Metabolome and microbiome signatures in the roots of citrus affected by huanglongbing. Phytopathology, 109(12), 2022-2032.

Ginnan, N. A., Dang, T., Bodaghi, S., Ruegger, P. M., Peacock, B. B., McCollum, G., ... & Borneman, J. (2018). Bacterial and fungal next generation sequencing datasets and metadata from citrus infected with ‘Candidatus Liberibacter asiaticus’. Phytobiomes, 2(2), 64-70.

Blacutt, A., Ginnan, N., Dang, T., Bodaghi, S., Vidalakis, G., Ruegger, P., ... & Roper, M. C. (2020). An in vitro pipeline for screening and selection of citrus-associated microbiota with potential anti-“Candidatus Liberibacter asiaticus” properties. Applied and environmental microbiology, 86(8), e02883-19.

Ginnan, N. A., Dang, T., Bodaghi, S., Ruegger, P. M., McCollum, G., England, G., ... & Roper, M. C. (2020). Disease-induced microbial shifts in citrus indicate microbiome-derived responses to huanglongbing across the disease severity spectrum. Phytobiomes Journal, 4(4), 375-387.

Zhou, Y., Tang, Y., Hu, C., Zhan, T., Zhang, S., Cai, M., & Zhao, X. (2021). Soil applied Ca, Mg and B altered phyllosphere and rhizosphere bacterial microbiome and reduced Huanglongbing incidence in Gannan Navel Orange. Science of the Total Environment, 791, 148046.

Penyalver, R., Roesch, L. F., Piquer-Salcedo, J. E., Forner-Giner, M. A., & Alguacil, M. D. M. (2022). From the bacterial citrus microbiome to the selection of potentially host-beneficial microbes. New Biotechnology, 70, 116-128.

Castellano-Hinojosa, A., Meyering, B., Nuzzo, A., Strauss, S. L., & Albrecht, U. (2021). Effect of plant biostimulants on root and plant health and the rhizosphere microbiome of citrus trees in huanglongbing-endemic conditions. Trees, 35(5), 1525-1539.

**Table S2.** Statistics of numbers of data with different health statuses and tissue sources collected for meta-analysis.

| **Health status** | **Tissue source** | **Data number** |
| --- | --- | --- |
| Health | Soil | 50 |
|  | Budwood | 3 |
|  | Leaf | 31 |
|  | Rhizosphere | 727 |
| HLB | Leaf | 380 |
|  | Budwood | 276 |
|  | Insect | 5 |
|  | Rhizosphere | 671 |

**Table S3.** Statistics of data numbers with meta information for meta-analysis after filtering.

| **Health status** | **Tissue source** | **Data number** |
| --- | --- | --- |
| Health | Leaf | 29 |
| Health | Rhizosphere | 207 |
| HLB | Leaf | 267 |
| HLB | Rhizosphere | 303 |

**Table S4.** Biomarkers with significant different abundances between HLB and healthy leaves.

| **Biomarkders** | **P value (t-test)** | **Enriched group** |
| --- | --- | --- |
| *Solanum melongena (eggplant)* | 1.47E-31 | Health |
| *Methylobacterium phyllosphaerae* | 2.55E-11 | Health |
| *Kosakonia cowanii* | 2.05E-22 | Health |
| *Pantoea agglomerans* | 5.28E-14 | Health |
| *Bacillus megaterium* | 5.56E-09 | Health |
| *Amnibacterium kyonggiense* | 7.04E-13 | Health |
| *Pantoea sp. PSNIH1* | 2.56E-20 | Health |
| *Acinetobacter soli* | 5.24E-16 | Health |
| *Stenotrophomonas maltophilia* | 3.91E-31 | Health |
| *Methylobacterium radiotolerans JCM 2831* | 2.29E-23 | Health |
| *Sphingomonas yunnanensis* | 6.27E-10 | Health |
| *Pantoea dispersa* | 6.83E-14 | Health |
| *Pseudomonas oryzihabitans* | 6.93E-25 | Health |
| *Microbacterium testaceum* | 1.64E-11 | Health |
| *Citrobacter werkmanii* | 1.78E-05 | Health |
| *Paraburkholderia rhizoxinica HKI 454* | 0.00042511 | HLB |
| *Candidatus Liberibacter asiaticus* | 8.53E-11 | HLB |

**Table S5.** Biomarkers with significant different abundances between HLB and healthy rhizosphere.

| **Biomarkers** | **P value (t-test)** | **Enriched group** |
| --- | --- | --- |
| *Pedomicrobium* | 6.62E-38 | Health |
| *RB41* | 1.74E-29 | Health |
| *Sphingomonas* | 2.77E-44 | Health |
| *Pir4 lineage* | 2.95E-37 | Health |
| *Nitrospira* | 1.49E-37 | Health |
| *Nocardioides* | 3.43E-31 | Health |
| *MND1* | 5.95E-37 | Health |
| *Mycobacterium* | 1.94E-21 | Health |
| *Bryobacter* | 3.62E-23 | Health |
| *mle1-7* | 1.14E-45 | Health |
| *Candidatus Udaeobacter* | 1.32E-27 | Health |
| *Ellin6055* | 1.60E-07 | Health |
| *Pseudonocardia* | 7.52E-22 | Health |
| *Pseudarthrobacter* | 2.31E-22 | Health |
| *Microvirga* | 6.21E-17 | Health |
| *Terrimonas* | 5.13E-24 | Health |
| *Microbacterium* | 4.00E-28 | Health |
| *Bradyrhizobium* | 3.77E-15 | Health |
| *Nordella* | 2.52E-23 | Health |
| *Ellin6067* | 5.82E-18 | Health |
| *Pirellula* | 3.25E-29 | Health |
| *Hirschia* | 9.45E-20 | Health |
| *Chryseobacterium* | 3.49E-13 | Health |
| *Parafilimonas* | 2.82E-13 | Health |
| *Xanthomonas* | 7.65E-41 | HLB |
| *Bacillus* | 4.18E-08 | HLB |
| *Burkholderia-Caballeronia-Paraburkholderia* | 2.45E-32 | HLB |
| *Streptomyces* | 1.38E-29 | HLB |

**Table S6.** PERMANOVA test for different factors contributing the variations in citrus microbiome.

| **Factors** | **R^2^** | **p-value** |
| --- | --- | --- |
| Batch | 0.240 | <0.001 |
| Variable region | 0.144 | <0.001 |
| Health status | 0.033 | <0.001 |
| Country | 0.021 | <0.001 |
| Continent | 0.017 | <0.001 |
| Platform | 0.005 | <0.001 |
| Tissue source | 0.003 | 0.015 |
